# Immortalization of primary marmoset skin fibroblasts by CRISPR-Cas9-mediated gene targeting

**DOI:** 10.1080/19768354.2022.2151509

**Published:** 2022-11-30

**Authors:** Yeon-Ju Jeong, Jeongin Cho, Jina Kwak, Young Hoon Sung, Byeong-Cheol Kang

**Affiliations:** a Convergence Medicine Research Center, Asan Institute for Life Sciences, Asan Medical Center, Seoul, Republic of Korea; b Department of Medical Science and Asan Medical Institute of Convergence Science and Technology, Asan Medical Center, University of Ulsan College of Medicine, Seoul, Republic of Korea; c Graduate School of Translational Medicine, Seoul National University College of Medicine, Seoul, Republic of Korea; d Department of Experimental Animal Research, Biomedical Research Institute, Seoul National University Hospital, Seoul, Republic of Korea; e Department of Convergence Medicine, University of Ulsan College of Medicine, Seoul, Republic of Korea; f Designed Animal Resource Center, Institute of GreenBio Science and Technology, Seoul National University, Pyeongchang-gun, Republic of Korea

**Keywords:** Marmoset, immortalization, cell line, Cas9, genotoxic stress

## Abstract

Immortalized cell lines can be used for diverse in vitro experiments, providing invaluable data before conducting in vivo studies *Callithrix jacchus*, the common marmoset, is a non-human primate model utilized for studying various human diseases. However, only a few immortalized marmoset cell lines are currently available. In the present study, we reveal that CRISPR-Cas9-mediated targeting of the *p53* gene or *CDKN2A* locus is an effective means for immortalizing primary marmoset skin fibroblasts. In addition to frameshift mutations that result in premature stop codons, in-frame mutations potentially destroying the DNA-binding motif of *p53* are frequently detected in immortalized cells. Like *Cdkn2a*-deficient mouse cells, *CDKN2A*-deficient marmoset cells express wild-type p53 proteins normally respond to genotoxic stresses, including adriamycin and etoposide. Taken together, these findings indicate that Cas9- mediated gene targeting of the *p53* gene or *CDKN2A* locus is an effective tool for establishing immortalized marmoset cell lines with defined genetic alterations.

## Introduction

The common marmoset, *Callithrix jacchus*, is an emerging non-human primate model used in neuroscience, immunology, reproductive science, and toxicology studies (Kishi et al. 2014). Despite the increasing demand for these animals, their supply is limited, as common marmoset is an endangered species (Malukiewicz et al. 2015). The supply of immortalized cell lines is unlimited, and their use for *in vitro* experiments before animal studies is an essential tool to minimize the number of *in vivo* studies sacrificing animals (Dumenco et al. 1995). However, only a limited number of immortalized marmoset cell lines are currently available (Guo et al. 2018; Petkov et al. 2018). This necessitates development of immortalized marmoset cell lines from specific tissues to be studied.

Because cell immortalization is a critical process for carcinogenesis, numerous immortalized cell lines have been isolated from various human cancers. Different from mouse embryonic stem (ES) cells (Han et al. 2020), normal diploid human cells eventually enter replicative senescence after a finite number of cell divisions (Hayflick 1965) and thus, to become immortalized, cells must escape replicative senescence, acquiring the capability of infinite cell division. Replicative senescence is attributable to shortening of telomeres, the ends of chromosomes, with each cell division (Blackburn 2000). Because normal somatic cells lack the telomerase activity necessary for telomere elongation, telomeres are gradually shortened by successive cell divisions, and finally, critically short telomeres trigger replicative senescence (Shay et al. 2001). Consistently, exogenous telomerase overexpression causes elongation of telomeres in primary diploid cells, which then actively proliferate, accompanied by suppressed expression of senescence biomarkers (Bodnar et al. 1998). Similarly, primary marmoset skin fibroblasts can be immortalized using the *human telomerase catalytic subunit (hTERT)* gene (Petkov et al. 2018).

*p53* is the most frequently mutated gene in human cancers, and approximately 90% of *p53* mutations are missense mutations in the DNA-binding domain (Baugh et al. 2018). *p53* mutations are common in spontaneously immortalized primary mouse embryonic fibroblasts (MEFs) (Harvey and Levine 1991), and overexpression of the mutant *p53* gene (del329) immortalizes normal human mammary epithelial cells (MECs) (Gao et al. 1996). Similarly, cellular senescence does not occur in *p53*-deficient primary MEFs (Harvey et al. 1993).

The *CDKN2A* locus, also known as *INK4A/ARF* locus alternatively encodes two tumor suppressor proteins, p16^INK4A^ and p14^ARF^ (p19^Arf^ in mice), and is one of the most frequently mutated genes in human cancers. Most mutations cause functional loss of both *p16^INK4A^* and *p14^ARF^* (Sharpless and DePinho 1999). In mice, genetic ablation of both *p16^Ink4a^* and *p19^Arf^* allows primary MEFs to consistently grow with no detectable senescent phase (Serrano et al. 1996). *p19^Arf^*-deficient MEFs with retention of *p16^Ink4a^* gene expression are also immortal (Kamijo et al. 1997), but immortalization of *p16^Ink4a^*-null MEFs is significantly lower than that of MEFs null for the *Cdkn2a* locus or *p19^Arf^* gene (Sharpless et al. 2001), indicating a predominant role for the *p19^Arf^* gene in cellular senescence.

In the present study, we determined if deficiencies in the *p53* gene or *CDKN2A* locus could immortalize primary common marmoset skin fibroblasts. To induce null mutations in these genes, a CRISPR-Cas9 system from *Streptococcus pyogenes* (Cho et al. 2013; Cong et al. 2013; Mali et al. 2013) was adapted and provided a simple and robust means for efficient immortalization of primary skin cells from common marmosets. This platform can potentially be used to establish immortalized cell lines that are essential for *in vitro* studies prior to *in vivo* experiments using common marmoset monkeys, which are available in very limited numbers due to their status as an endangered species.

## Materials and methods

### Animal, biopsy, and primary skin cell culture

A 3-year-old female common marmoset was purchased from laboratory animal supplier, CLEA (Kawasaki, Japan). Animal procedures were approved by the Institutional Animal Care and Use Committee of the Biomedical Research Institute at the Seoul National University Hospital, an accredited research institute of the Association for Assessment and Accreditation of Laboratory Animal Care International (AAALAC; SNUH-IACUC No: 20-0161). An abdominal skin sample was collected during autopsy. Primary skin fibroblasts were isolated as described previously (Seluanov et al. 2010). Briefly, 7 days after digestion of skin tissues using Liberase^TM^ (Roche) in DMEM/F12 (Gibco), cells were transferred to EMEM (ATCC) containing 10% (v/v) FBS (Sigma-Adrich) and 10 unit/ml Penicillin/Streptomycin. Upon confluence, cells were detached using 0.05% Trypsin-EDTA (Invitrogen) and were then cultivated with 10% (v/v) FBS/DMEM media supplemented with Penicillin/Streptomycin.

### Cloning of single guide RNAs (sgRNAs) and establishment of clonal cell lines

sgRNAs specific for marmoset *p53* gene and *CDKN2A* locus were designed using Benchling (https://www.benchling.com), and the following oligomers were purchased from Macrogen, Inc.: 5′–CACCGTGTAACAGTTCCTGCATGGG–3′ and 5′–AAACCCCATGCAGGAACTGTTACAC–3′ for *p53*-specific sgRNA; 5′–CACCGACCGGTTCACGACGCTGCCC–3′ and 5′–AAACGGGCAGCGTCGTGAACCGGTC–3′ for *CDKN2A* locus-specific sgRNA. For the delivery of CRISPR-Cas9 into the cells, a lentiCRISPRv2 vector, a gift from Feng Zhang (Addgene plasmid #52961; http://n2t.net/addgene:52961; RRID:Addgene_52961), was used. Each pair of oligomers was annealed and cloned into lentiCRISPRv2 using BsmBI restriction enzymes from New England BioLabs, Inc. (NEB). Infectious lentiviral particles were produced as described previously (Sung et al. 2013). After lentivirus infection, cells were treated with 2 μg/ml puromycin (Sigma-Aldrich) to remove non-infected cells, and puromycin-resistant cells were used to establish clonal cell lines.

### Genotype analysis

To determine the presence of indel mutations in pooled or clonal cells, genomic DNA samples were isolated and T7 endonuclease I (T7E1) assays were conducted as previously described (Oh et al. 2021). Briefly, genomic regions encompassing the target site were PCR-amplified, heteroduplex DNAs were generated by heat denaturation and reannealing, and products were purified using a PCR cleanup kit from iNtRON Biotechnology, Inc (Seongnam, Korea). After treatment with 5 units T7E1 (NEB) for 15 min at 37°C, samples were analyzed by agarose gel electrophoresis. The following oligomers were used for PCR amplification: 5′–TTGACCTCCCCATAATTCCA–3′ and 5′–TCGTCCTTTCTGGAGCCTAA–3′ (509 bp) for the *TP53* gene; 5′–TTGTCCTTGTCTCACAGGGC–3′ and 5′–CTGATGATTGGCGCGATTGG–3′ (696 bp) for the *CDKN2A* locus. For Sanger sequencing, PCR products were subcloned using a T-blunt TA cloning kit (SolGent Co., Ltd., Seoul, Korea), and miniprep DNA samples were then prepared using a DNA-spin™ Plasmid DNA Purification Kit (iNtRON Biotechnology, Inc., Seongnam, Korea) and were sequenced at Macrogen (Seoul, Korea).

The following oligomers were used for PCR amplification for the *CDKN2B* locus: 5′–CCAGACACCGGAGACTTGAA–3′ and 5′–CCCCTTTTGCAGCCTTCATC–3′ (498 bp). For Sanger sequencing, PCR products were purified and sequenced using the following oligomer: 5′–GACTTGAACACCTCTGCACTG–3′.

### In vitro growth and senescence-associated β-galactosidase (SAβ-gal) assays

To measure cumulative population doublings, cells were plated at 3 × 10^5^ cells/60 mm dish or 1 × 10^6^ cells/100 mm dish. Confluent cultures were trypsinized, viable cells were counted, and cells were replated at the same density. For viable cell counting, aliquots of trypsinized cells were stained with Trypan Blue staining solution (Invitrogen) and were counted using a Countess II cell counter (Invitrogen) equipped with a Countess^TM^ cell counting chamber slide (Invitrogen). If tissue cultures were not confluent, cells were grown until confluence after adding fresh media. To count senescent cells, SAβ-gal assays were conducted using a Senescence β-Galactosidase Staining Kit (Cell Signaling) according to the manufacturer’s instructions (Kim et al. 2020). Photographs were taken using a Nikon eclipse Ts2 microscope (Nikon, Tokyo, Japan) equipped with an HK3.1 CMOS digital camera and HKBASIC Software (KOPTIC).

### Semi-quantitative reverse transcription (RT)-polymerase chain reaction (RT-PCR)

Total RNAs were isolated using TRIzol Reagent (Thermo Fisher Scientific, Waltham, MA) according to the manufacturer’s instructions, and the SuperScript III First-strand Synthesis System (Invitrogen, Waltham, MA) was used for reverse transcription (RT). Semi-quantitative RT-PCR reactions were conducted using RT products as template and the following primers: 5′–GATCTGCCTTCCCAAAGGGC–3′ and 5′–AGTGAGGCATTAGGGTGCAA–3′ for *p21^WAF1^* gene (125 bp, XM_035294655); 5′–ACCCTGTGCAAGACCTACAG–3′ and 5′–TGGCGTTTTCTTTGCCGTTC–3′ for *MDM2* gene (142 bp, XM_009004180); 5′–GCTGCTCACCTCTGGTGCCA–3′ and 5′–TTCAATCGGGGACGTCTGAG–3′ for *p14^ARF^* gene (583 bp, XM_035306720); and 5′–TCGGAGTCAACGGATTTGGTC–3′ and 5′–TTCCCGTTCTCAGCCTTGAC–3′ for *GAPDH* gene (181 bp, XM_035255536); 5′– CAGAGCTAAAGGAGCTGCTGACC–3′ and 5′–CCTGGAAGTCCACCTCGTTGTC–3′ for *S100A4* gene (126 bp, XM_035279100); 5′–CTGGATTCACTCCCTCTGGTTG –3′ and 5′–CATGATGCTGAGAAGTTTCGTTG –3′ for *VIM* gene (106 bp, XM_002750069); 5′–AGAAAGTGGATGCCGCCTTTAA–3′ and 5′–CATTCCAGGCATCCGCGATGAG –3′ for *MMP2* gene (138 bp, XM_017966916); 5-GCCACTCCTACAACCAATATTCTC–3′ and 5′–GCTTGTTCCTCTGGGTTGGAAAG–3′ for *FN1* gene (147 bp, XM_008999454).

### Western blot analysis

Cells were treated with adriamycin (Sigma-Aldrich) or etoposide (Sigma-Aldrich), and SDS protein samples were then prepared by directly lysing the cells in SDS sample buffer (Elpisbiotech. Inc., Daejeon, Korea) after washing with ice-cold phosphate-buffered saline (PBS).

Western blots were conducted using antibodies against MDM2 (Santa Cruz Biotechnology, Inc., #sc-965), p21^WAF1^ (Santa Cruz Biotechnology, Inc., #sc-6246), p53 (Santa Cruz Biotechnology, Inc., #sc-126), p16^INK4A^ (Santa Cruz Biotechnology, Inc., #sc-1661), p14^ARF^ (Cell Signaling #74560), and GAPDH (Cell Signaling #2118).

## Results

### Targeted inactivation of the p53 gene in primary marmoset skin fibroblasts using CRISPR-Cas9

**Figure 1.**
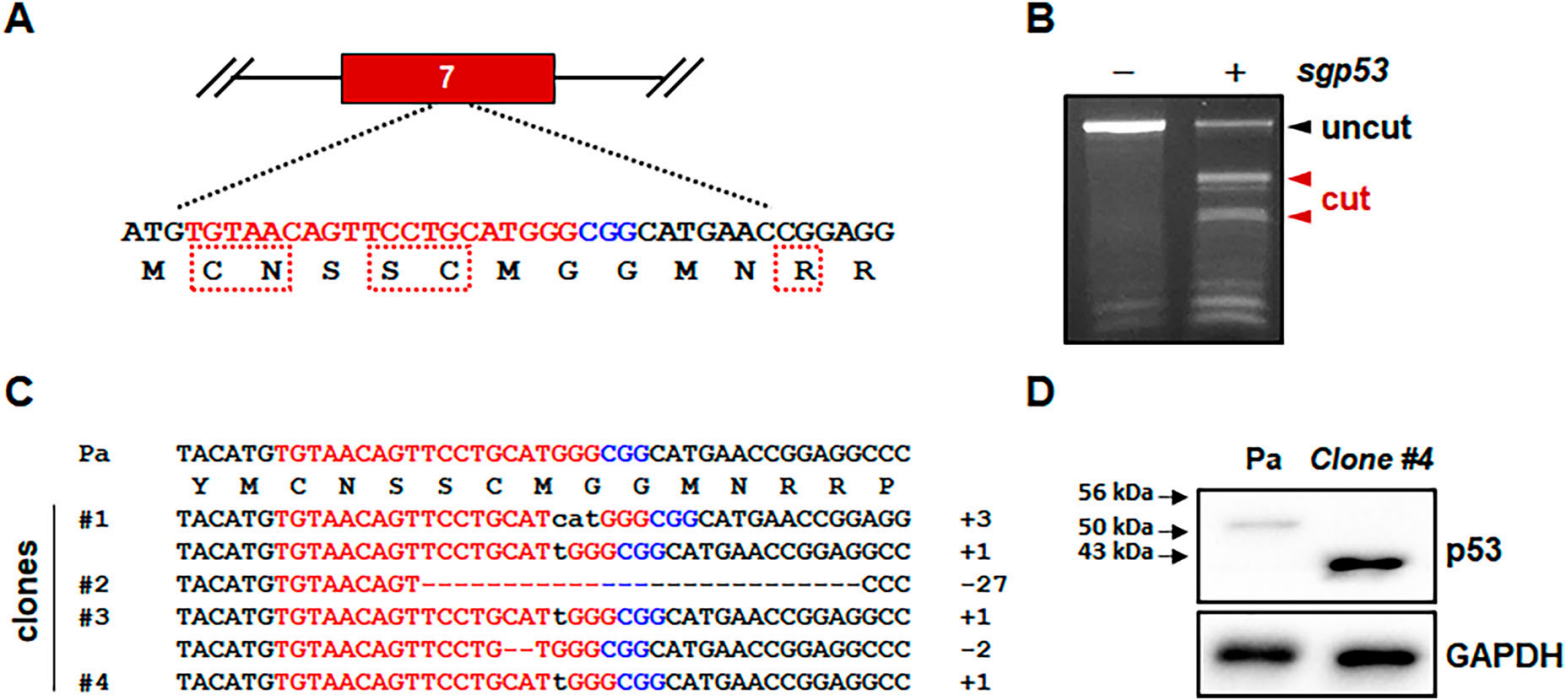
Cas9-mediated *p53* gene targeting in primary skin fibroblasts isolated from a common marmoset monkey. (A) Schematic representation of the *p53*-specific sgRNA on exon 7. Target and PAM sequences are denoted by red and blue colors, respectively. Dotted boxes indicate the amino acids critical for the DNA-binding function of p53 protein as a transcription factor. (B) Endonuclease activity induced by *p53*-specific sgRNA (*sgp53*). After infection with lentivirus expressing both Cas9 and *p53*-specific sgRNA, Cas9-induced indel mutations was examined using genomic DNA samples from parental primary skin fibroblasts (−) and puromycin-selected cells (+) by T7E1 assay. (C) Mutated *p53* sequences observed in the mutant cell clones. − denotes deleted nucleotides; sequences in lower case denote nucleotide insertions. (D) Western blot analysis of p53 proteins in primary skin fibroblasts (parental; Pa) and *p53^-/-^* skin fibroblasts (clone #4 in c).

Because most mutations of the p53 DNA-binding domain cause reduction or complete loss of p53 transcriptional activity (Baugh et al. 2018), we designed a sgRNA specific for exon 7, which encodes critical amino acid residues of the p53 protein DNA-binding domain (Figure 1(A)). We could not find any potential off-target sites with less than three mismatches from the sgRNA on the marmoset genome. When this sgRNA was expressed with Cas9 using a lentiviral vector, indel mutations were robustly induced in primary marmoset skin fibroblasts (Figure 1(B)). As polyclonal marmoset skin fibroblasts actively proliferated and contained diverse indel mutations (Figure S1), we established multiple clonal cell lines with in-frame and/or frameshift mutations (Figure 1(C)).

Among the *p53*-mutated clones, we selected mutant clone #4, which was homozygous for a 1 bp insertion in the target sequence (Figure 1(C)). Because of the frameshift mutation, clone #4 expressed a mutant p53 protein comprised of ∼1–244 amino acid residues of wild-type marmoset p53 protein (395 amino acid residues, XM_002747948) and 20 aberrant amino acid residues. Accordingly, while Western blot analysis detected a wild-type p53-specific signal in parental primary skin fibroblasts, *p53* mutant cells strongly expressed a smaller mutant p53 protein that is approximately 43 kDa, but not wild-type p53 protein (Figure 1(D)).

### Targeted mutations of the CDKN2A locus in primary marmoset skin fibroblasts using CRISPR-Cas9

**Figure 2.**
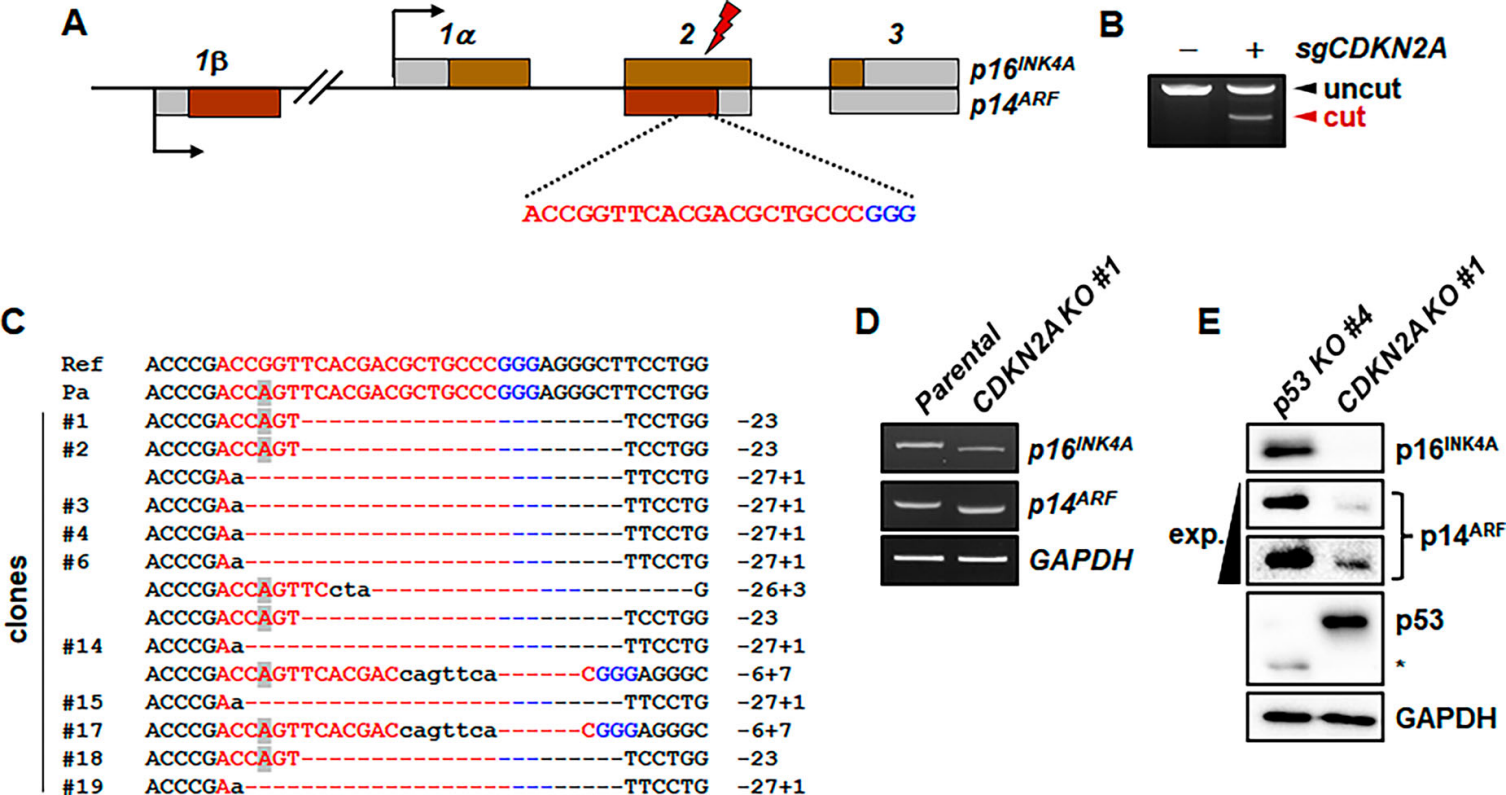
Generation of mutant marmoset skin fibroblasts deficient in both *p16^INK4A^* and *p14^ARF^* genes using CRISPR-Cas9. (A) Schematic representation of *CDKN2A*-specific sgRNA on exon 2, which is common to both *p16^INK4A^* and *p14^ARF^*. Target and PAM sequences are denoted by red and blue colors, respectively. (B) Endonuclease activity of Cas9 induced by common exon 2-specific sgRNA (*sgCDKN2A*). T7E1 assays were conducted using genomic DNA samples from parental primary skin fibroblasts (−) and puromycin-selected cells (+) after infection with lentivirus expressing both Cas9 and *sgCDKN2A*. (C) Mutated *CDKN2A* sequences observed in the clones. A polymorphic nucleotide is shaded in gray. Ref, reference genomic DNA sequence of the *CDKN2A* locus; Pa, parental marmoset skin fibroblasts; − denotes deleted nucleotides; sequences in lower case denote nucleotide insertions. (D) Western blot analysis of p16^INK4A^ and p14^ARF^ proteins in *p53^-/-^* cells (Figure 1 clone #4) and *CDKN2A^-/-^* cells (clone #1 in c). Short exp., short exposure time; long exp., long exposure time.

The *CDKN2A* locus encodes two proteins, p16^INK4A^ and p14^ARF^, with exon 1 sequences, 1α and 1β, respectively, and alternative reading frames of the common exons in humans and mice (Quelle et al. 1995). The *CDKN2A* locus in the marmoset genome also encodes the *p16^INK4A^* gene (Figure 2(A)). Although marmoset p14^ARF^ protein and transcript have not yet been annotated, the marmoset genome harbors a putative exon 1β that is upstream of exon 1α (Figure 2(A)). Accordingly, the predicted transcript variant X2 (XM_035306720), which harbors putative exon 1β, alternatively encodes the putative marmoset p14^ARF^ protein (Figure S2A), which is highly homologous to human p14^ARF^ protein (Figure S2B).

To inactivate both the *p16^INK4A^* and *p14^ARF^* genes simultaneously, we selected an sgRNA specifically targeting the common exon 2 (Figure 2(A)) and confirmed that the sgRNA actively induced indel mutations in primary marmoset skin fibroblasts (Figure 2(B)). The sgRNA has an off-target site with a 1-bp mismatch on the highly homologous *CDKN2B* gene. Except for this off-target site, there is no potential off-target sites with less than three mismatches from our *CDKN2A* gene-specific sgRNA on the marmoset genome (Tsai et al. 2015; Kim et al. 2016). The *CDKN2A*-targeted marmoset skin fibroblasts were used to establish clonal cells deficient for both *p16^INK4A^* and *p14^ARF^*. We detected a sequence variation, XM_035306720 c.286G > A (p.Gly96Ser), which was prevalent in our wild-type marmosets (Figure S3). However, the 1 bp mismatch between the target sequence and the sgRNA did not compromise the action of CRISPR-Cas9, and thus most of the clones had both insertions and deletions resulting in simultaneous frameshifts in the *p16^INK4A^* and *p14^ARF^* genes (Figure 2(C)). For example, the 23 bp-deleted clone #1 encoded mutant p16^INK4A^ protein was composed of 76 amino acid residues of wild-type p16^INK4A^ protein at the N-terminus and 28 amino acid residues of p14^ARF^ protein at the C-terminus (Figure S4A), reflecting the alternative reading frame in the *CDKN2A* locus. For the *p14^ARF^* gene, the frameshift mutation of clone #1 involved the third reading frame that was not used by either the *p16^INK4A^* or *p14^ARF^* genes, and thus the open reading frame encoded a mutant p14^ARF^ protein containing 97 amino acid residues of wild-type p14^ARF^ protein at the N-terminus and 103 mutated amino acid residues at the C-terminus (Figure S4B).

We confirmed expression of *p16^INK4A^* and *p14^ARF^* in *CDKN2A*-mutant clone #1. When compared with those of parental cells, transcription of the *p16^INK4A^* gene was slightly decreased, while transcription of the *p14^ARF^* gene was unchanged (Figure 2(D)). Due to the 23 bp deletions in *CDKN2A*-mutant clone #1, the sizes of the *p16^INK4A^* and *p14^ARF^* cDNAs were slightly smaller than those of the parental cells (Figure 2(D)). We also measured expression of p16^INK4A^ and p14^ARF^ proteins in the *CDKN2A*-mutant cells using *p53*-mutant clone #4 as a positive control (Figure 2(E)). p16^INK4A^ protein was robustly expressed in *p53*-mutant cells, but not in *CDKN2A*-mutant cells (Figure 2(E)). Although a weak non-specific signal was present, *p53*-mutant cells robustly expressed p14^ARF^ protein, but *CDKN2A*-mutant cells did not (Figure 2(E)).

We also monitored the off-target mutation of the *CDKN2B* gene (Figure S5). The sequencing result showed the co-existence of wild-type and 9-bp deleted mutant alleles (Figure S5).

### Characterization of p53- and CDKN2A-mutant marmoset cells

**Figure 3.**
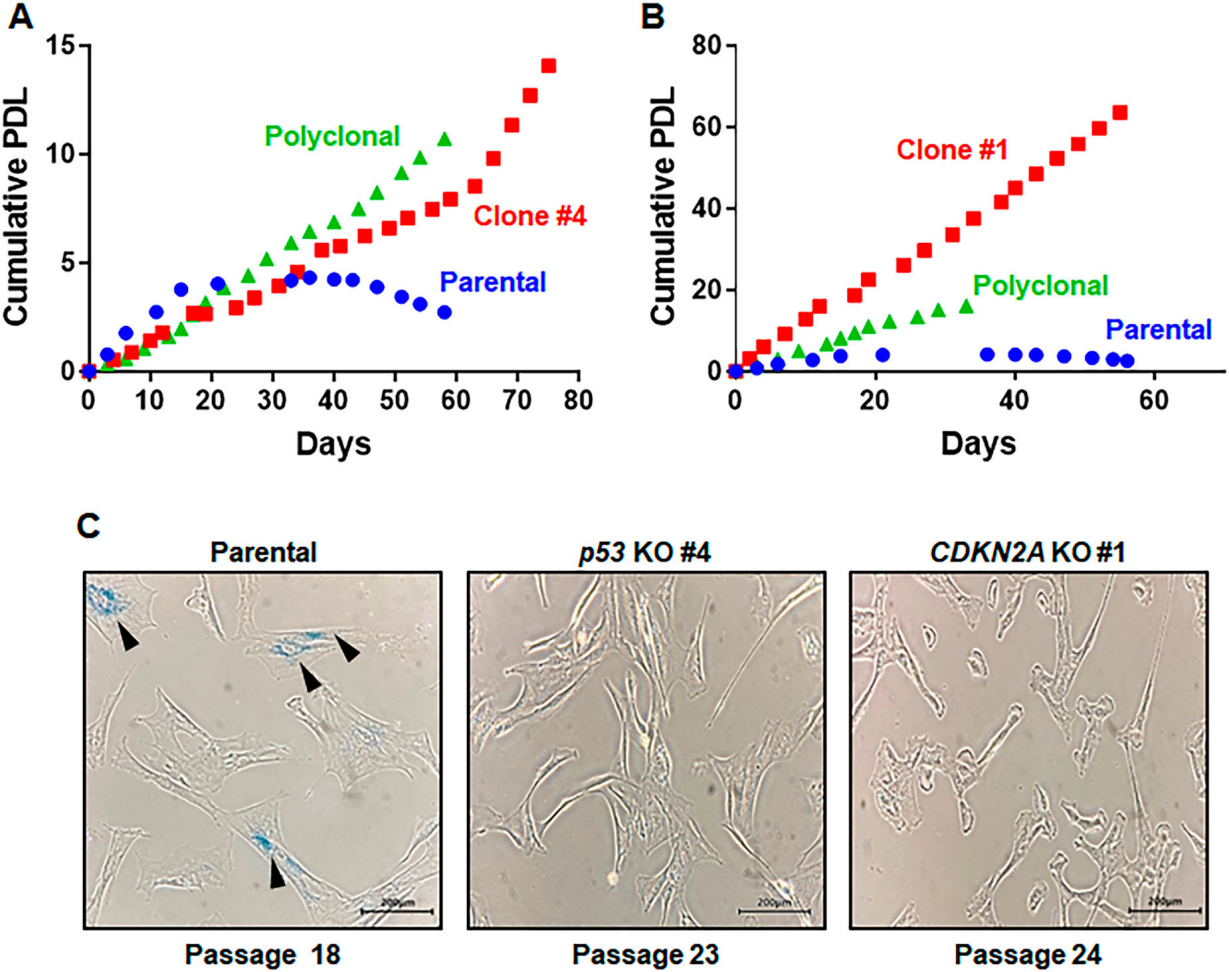
Immortalized phenotypes observed in both *p53*- and *CDKN2A*-deficient marmoset skin fibroblasts. (A, B) Cumulative population doublings (PDs) of *p53*-deficient (A) and *CDKN2A*-deficient (B) marmoset skin fibroblasts. Growth of polyclonal cells (purple triangles) and monoclonal mutant cells (red rectangles; *p53^-/-^* clone #4 and *CDKN2A^-/-^* clone #1) was compared with that of parental primary skin fibroblasts (blue circle). (C) Senescence-associated β-galactosidase (SAβ-gal) assays conducted with marmoset skin fibroblasts and immortalized *p53*- and *CDKN2A*-deficient clones at the denoted passages. Scale bar, 200 μm.

We examined whether *p53*- and *CDKN2A*-mutant marmoset cells continued to divide without developing senescence. Cumulative population doubling levels (PDLs) of mutant marmoset cells were measured and compared with that of primary marmoset cells (Figure 3(A,B)). The proliferation of primary cells gradually slowed and eventually ceased (Figure 3(A,B)). For *p53*-mutant marmoset cells, both polyclonal and monoclonal cells continuously proliferated without decreased growth rates and had similar growth characteristics (Figure 3(A)). *p53*-mutant cells (clone #4) were thawed at passage 8 and cell growth was measured from passage 10 to passage 33, resulting in 17 cumulative population doublings (PDs). For *CDKN2A-*mutant cells, the growth rate of polyclonal cells did not decrease (Figure 3(B)). Compared with polyclonal cells, *CDKN2A*-mutant clone #1 exhibited an increased growth rate (Figure 3(B)) with 63 cumulative PD when cell growth was measured from passage 18 to passage 35. *CDKN2A-*mutant cells retained higher proliferation capacities than those of *p53*-mutant cells (Figure 3(A,B)). Most parental cells were enlarged and flattened by passage 18 compared with passage 8, a characteristic feature of senescent cells. In addition, *p53* and *CDKN2A*-mutant cells did not lose their fibroblast characters (Figure 3(C) and Figure S6). Notably, senescence-associated β-galactosidase (SA-βgal)-positive cells were frequently present among primary marmoset cells at passage 18, but not in *p53* (passage 23)- and *CDKN2A* (passage 24)- mutant clones (Figure 3(C)). Taken together, these data support establishment of genetically defined immortalized marmoset cell lines.

**Figure 4.**
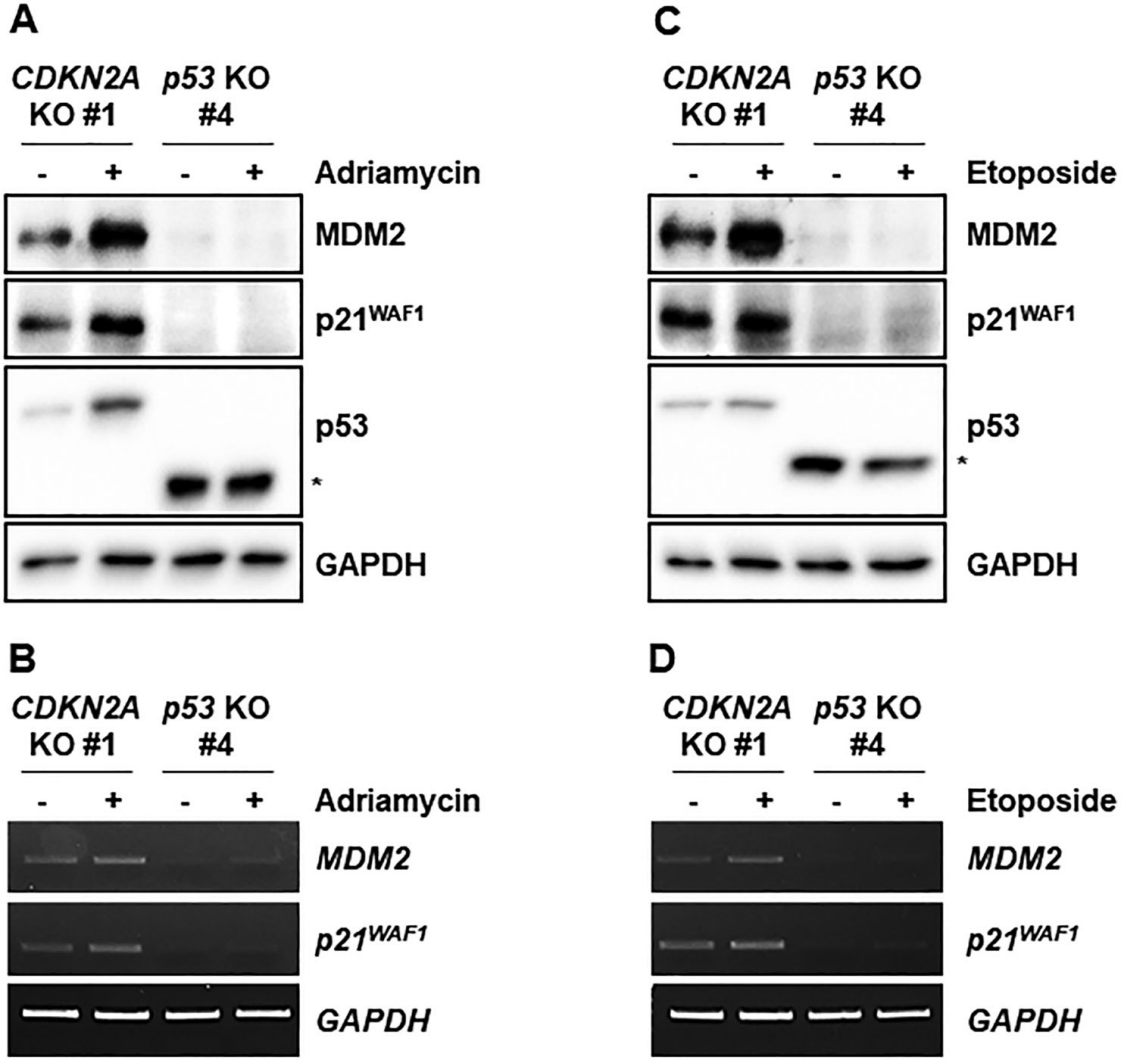
Induction of p53 target genes by genotoxic stresses in immortalized *CDKN2A*-deficient marmoset skin fibroblasts. (A) Western blot analysis of p53 and its target gene products, MDM2 and p21^WAF1^, in immortalized *p53*- and *CDKN2A*-deficient marmoset skin fibroblasts treated with 200 ng/ml adriamycin for 4 h. (B) Assessment of adriamycin-induced p53 target gene (*MDM2* and *p21^WAF1^*) transcription by semi-quantitative RT-PCR. Immortalized marmoset cells were treated as in a. The asterisk indicates truncated marmoset p53 proteins. (C) Western blot analysis of p53 and its target gene products, *MDM2* and *p21^WAF1^*, in immortalized *p53*- and *CDKN2A*-deficient marmoset skin fibroblasts treated with 0.5 μM etoposide for 4 h. The asterisk indicates truncated marmoset p53 proteins. (D) Etoposide induction of p53 target gene (*MDM2* and *p21^WAF1^*) transcription in immortalized marmoset cells. Semi-quantitative RT-PCR reactions were conducted with cells treated as in c.

Genotoxic stresses activate p53 and would thus be expected to increase expression of p53 target genes in the *CDKN2A*-mutant cell line with intact *p53*. We treated *CDKN2A*- and *p53*-mutant cell lines with adriamycin and etoposide and measured expression of p53 and its target genes (Figure 4). Adriamycin stabilized p53 protein and increased MDM2 and p21^WAF1^ protein levels in the *CDKN2A*-mutant marmoset cell line, which did not occur in the *p53*-mutant marmoset cell line (Figure 4(A)). Transcript levels of p53 target genes *MDM2* and *p21^WAF1^* were concomitantly increased in the *CDKN2A*-mutant cell line, but not in the *p53*-mutant cell line (Figure 4(B)). Consistently, etoposide treatment also increased p53 protein levels and target gene expressions (Figure 4(C,D)).

Cumulatively, these findings demonstrated that CRISPR-Cas9-mediated knockout of the *p53* gene and *CDKN2A* locus is a simple means to establish immortalized cell lines from marmoset skin biopsies. In turn, the resulting combination of *p53*-proficient (*CDKN2A*-mutant) and *p53*-deficient cells is an excellent *in vitro* model for the study of genotoxic stresses.

## Discussion

Immortalized human cell lines are essential for *in vitro* experiments and have contributed enormously to our understanding of the cellular and biochemical mechanisms underlying biological phenomena and pathological conditions. Common marmosets are a versatile non-human primate model suitable for studying a broad range of human diseases and generating gene-edited animal models. Because extensive *in vitro* experiments should be conducted prior to *in vivo* experiments using live marmosets due to limited animal availability and ethical considerations, immortalized cell lines derived from marmoset tissues of interest will greatly enhance the efficiency of these *in vitro* studies.

Recently, several reports have described successful generation of immortalized marmoset cell lines, and our study will provide an alternative and complementary method (Guo et al. 2018; Petkov et al. 2018; Orimoto et al. 2022). As in normal human cells that undergo a finite number of cell divisions (Bodnar et al. 1998), piggyBac transposon-mediated expression of the *hTERT* gene successfully immortalizes marmoset skin fibroblasts (Petkov et al. 2018). By contrast, *hTERT* expression alone is not sufficient for immortalization of marmoset muscle fibroblasts; a mutant form of *cyclin-dependent kinase 4* (*CDK4^R24C^*) and overexpression of wild-type *Cyclin D1* are also required (Orimoto et al. 2022). *CDK4^R24C^* mutation predisposes humans to hereditary melanoma and abolishes the ability of CDK4 protein to bind p16^INK4A^ protein (Wolfel et al. 1995; Zuo et al. 1996). Therefore, it can be reasonably expected that the *p16^INK4A^*-mediated senescence signal was perturbed by expression of CDK4^R24C^ in the immortalized marmoset muscle fibroblasts. Based on our results, *CDKN2A* gene knockout could exert a comparable effect to introducing the *CDK4^R24C^* transgene.

As previously reported in human-induced hepatocytes (hiHeps) (Huang et al. 2014), the simian virus 40 (SV40) large T-antigen also immortalizes marmoset hepatic progenitor cells (Guo et al. 2018). SV40 large T-antigen is effective for cellular immortalization but inactivates the *p53* gene (Ahuja et al. 2005). This suggests that SV40 large T-antigen-immortalized cells would not be suitable for study of *p53*-dependent cellular responses. Therefore, marmoset cells immortalized by *CDKN2A* gene targeting are preferable, as these cells retain functional *p53*. In support of this, we demonstrated that genotoxic stress-induced gene expression could be analyzed using *CDKN2A*- and *p53*-deficient immortalized cells as a pair.

The present study contributes to the development of novel marmoset cell lines from tissue types of interest. Our strategy can be improved by avoiding the off-target mutation detected in the highly homologous *CDKN2B* gene (Figure S5). It will be accomplished by targeting the exon 1β to generate *p14^ARF^*-deficient but *p16^INK4A^*-proficient cell lines as *p19^Arf^* deficiency alone makes primary MEFs immortal (Kamijo et al. 1997). In addition, vector-free or adeno-associated virus (AAV) vector systems are suitable for minimizing off-target effects induced by the constitutive expression of the CRISPR-Cas9 system. These cell lines will expedite biomedical studies using common marmoset monkeys and minimize the scale of animal experiments by allowing conduct of extensive *in vitro* experiments prior to initiating *in vivo* studies.

## Supplementary Material

Supplemental Material
