# Supplementary material for: Immortalization of primary marmoset skin fibroblasts by CRISPR-Cas9-mediated gene targeting
Source: Anim Cells Syst (Seoul). 2022 Nov 30;26(6):266–74. doi: 10.1080/19768354.2022.2151509 (PMC9809370; doi:10.1080/19768354.2022.2151509)
Supplement: Supplemental Material [file TACS_A_2151509_SM7774.pdf]

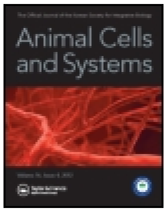

Immortalization of Primary Marmoset Skin Fibroblasts by CRISPR-Cas9-mediated Gene Targeting

Journal:	*Animal Cells and Systems*
Manuscript ID	TACS-2022-0150.R1
Manuscript Type:	Research Article
Keywords:	Marmoset, Immortalization, Cell line, Cas9, Genotoxic stress

SCHOLARONE™
Manuscripts

```
ACCATTCACTACAACTACATGTGTAACAGTTCCTGCATGGGCGGCATGAACCGGAGGCCCATCCTCACCATCATCACTCTGGAAGACTCC    WT
 T  I  H  Y  N  Y  M  C  N  S  S  C  M  G  G  M  N  R  R  P  I  L  T  I  I  T  L  E  D  S
ACCATTCACTACAACTACATGTGTAACAGTTCCTGCA-----------------------------------------------------    -172
gagccacat---(-56)--------------------------GCATGAACCGGAGGCCCATCCTCACCATCATCACTCTGGAAGACTCC    -56+8
ACCATTCACTACAACTACATGTGTAACAGT---------------------------CCCATCCTCACCATCATCACTCTGGAAGACTCC    -27
ACCATTCACTACAACTACATGTGTAAC--------------------------CGGAGGCCCATCCTCACCATCATCACTCTGGAAGACTCC    -24
ACCATTCACTACAACTACATGTGTAACAGTTCC---------GGCATGAACCGGAGGCCCATCCTCACCATCATCACTCTGGAAGACTCC    -9
ACCATTCACTACAACTACATGTGTAACAGTTCCTGCAT--------TGAACCGGAGGCCCATCCTCACCATCATCACTCTGGAAGACTCC    -8
ACCATTCACTACAACTACATGTGTAACAGTTCCTGCATcatGGGCGGCATGAACCGGAGGCCCATCCTCACCATCATCACTCTGGAAGAC    +3
ACCATTCACTACAACTACATGTGTAACAGTTCCTGCATtGGGCGGCATGAACCGGAGGCCCATCCTCACCATCATCACTCTGGAAGACTC    +1
```

Figure S1 Mutated *p53* sequences observed in puromycin-selected marmoset cells. Primary marmoset skin fibroblasts were infected with lentiviral particles expressing Cas9 and *p53*-specific sgRNA, infected cells were selected with puromycin, and indel mutations were detected by Sanger sequencing. Target and PAM sequences are denoted by red and blue colors, respectively. – denotes deleted nucleotides; sequences in lower case denote nucleotide insertions.

For Peer Review Only

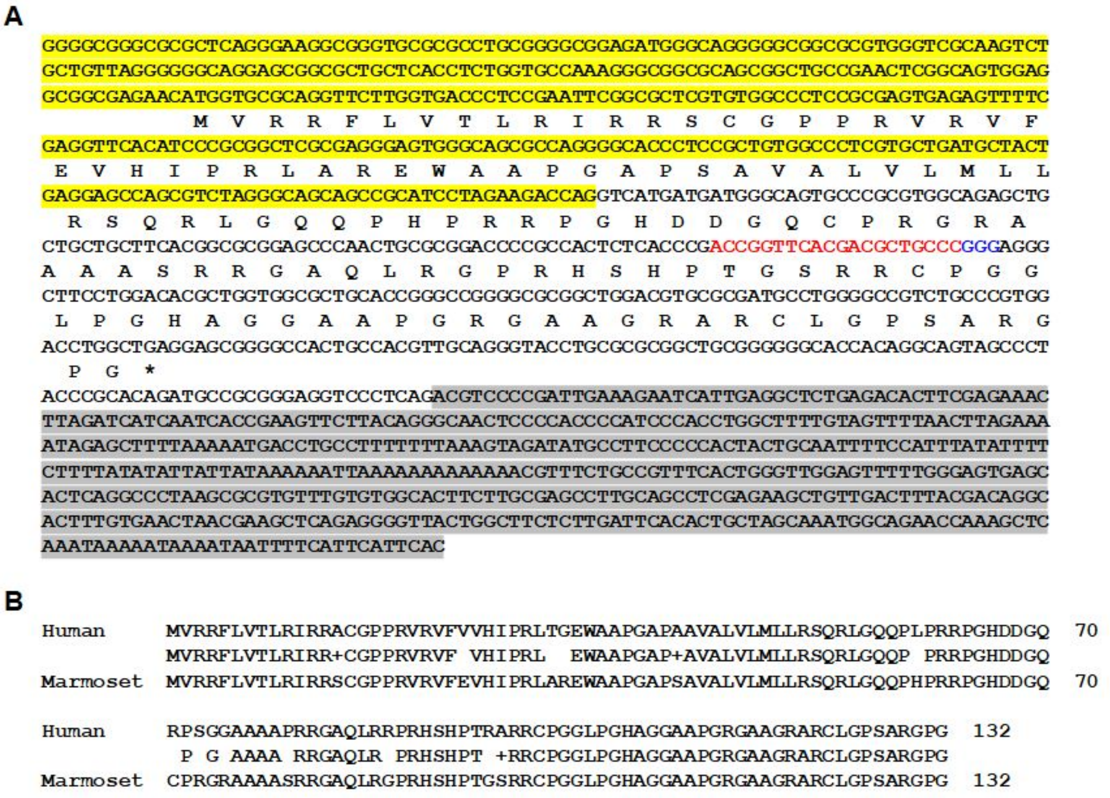

Figure S2 Deduced amino acid sequence of the marmoset p14^{ARF} protein and the sequence alignment to human p14^{ARF}. (A) Amino acid sequence of marmoset p14^{ARF} protein deduced from transcript variant X2 (XM_035306720). Exon 1β is shaded in yellow, and the common exon 3 is shaded in gray. sgRNA and PAM sequences are denoted in red and blue, respectively. (B) Comparison of human and marmoset p14^{ARF} proteins. The marmoset p14^{ARF} protein was aligned to the amino acid sequence of its human orthologue (NP_478102.2) using protein BLAST software.

Review Only

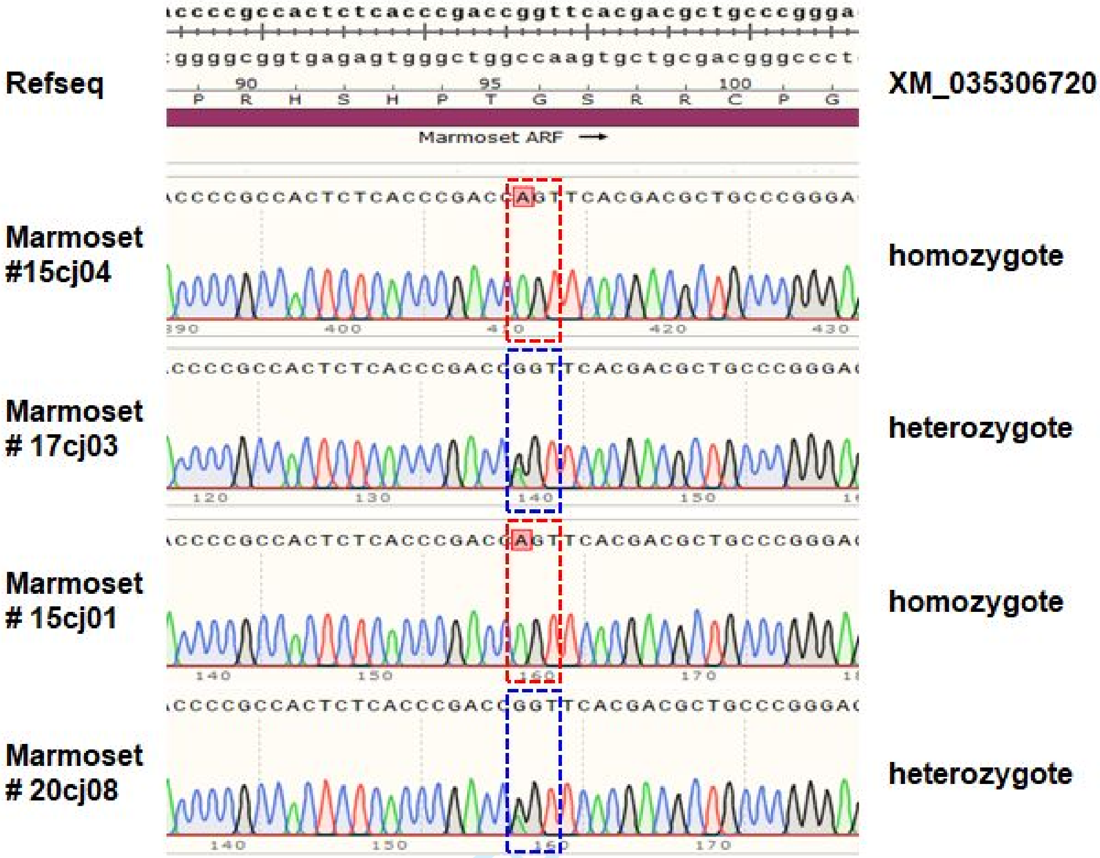

Figure S3 The DNA sequence polymorphism XM_035306720 c.286G>A at the marmoset *CDKN2A* locus. The DNA sequences of exon 2 were analyzed using four genomic DNA samples obtained from the primary marmoset skin cells (#15cj04) used to establish immortalized cells and three animals (#17cj03, #15cj01 and #20cj08) randomly selected from our marmoset colony. These nucleotide sequences were compared with the reference DNA sequence (RefSeq) using SnapGene software (GSL Biotech LLC). Polymorphic DNA regions are indicated by dashed boxes. Homozygotes are shown in red and heterozygotes are shown in blue.

URL: http://mc.manuscriptcentral.com/tacs Email: TACS-peerreview@journals.tandf.co.uk

A

```
      WT MEPSADWLATAAARGRVEEVRALLEAGAPPNAPNSYGRRPIQVMMMGSARVAELLLLHGA    60
Clone #1 MEPSADWLATAAARGRVEEVRALLEAGAPPNAPNSYGRRPIQVMMMGSARVAELLLLHGA    60

      WT EPNCADPATLTRPVHDAAREGFLDTLVALHRAGARLDVRDAWGRLPVDLAEERGHCHVAG   120
Clone #1 EPNCADPATLTRPVPGHAGGAAPGRGAAGRARCLGPSARGPG                     102

      WT YLRAAAGGTTGSSPTRTDAAGGPSDVPD                                   148
```

B

```
      WT MVRRFLVTLRIRRSCGPPRVRVFEVHIPRLAREWAAPGAPSAVALVLMLLRSQRLGQQPH    60
Clone #1 MVRRFLVTLRIRRSCGPPRVRVFEVHIPRLAREWAAPGAPSAVALVLMLLRSQRLGQQPH    60

      WT PRRPGHDDGQCPRGRAAAASRRGAQLRGPRHSHPTGSRRCPGGLPGHAGGAAPGRGAAGR   120
Clone #1 PRRPGHDDGQCPRGRAAAASRRGAQLRGPRHSHPTGSWTRWWRCTGPGRGWTCAMPGAVC   120

      WT ARCLGPSARGPG                                                   132
Clone #1 PWTWLRSGATATLQGTCARLRGAPQAVALPAQMPREVPQTSPIERIIEALRHFEKLRSSI   180

Clone #1 TEVLTGQLPTPSHLAFVVLT                                           200
```

Figure S4 Amino acid sequences deduced from the mutant allele of *CDKN2A*$^{-/-}$ cells (clone #1). Mutant amino acid sequences were deduced and aligned to the wild-type amino acid sequences of p16^{INK4A} (A) and p14^{ARF} (B) proteins. The amino acids in red indicate those resulting from the frameshift mutation in immortalized cells.

For Peer Review Only

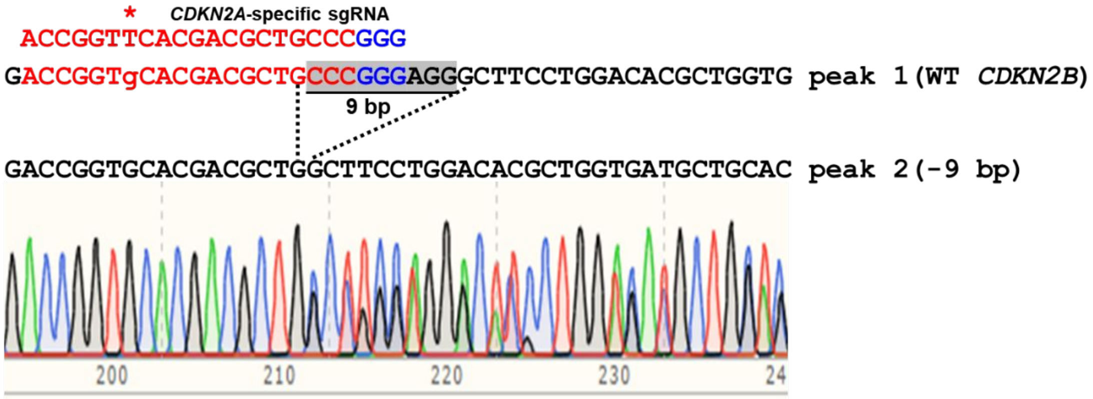

Figure S5 The Sanger sequencing result showing the off-target mutations in the *CDKN2B* gene of *CDKN2A*$^{-/-}$ clone #1. Compared to the *CDKN2A*-specific sgRNA, the off-target site on the *CDKN2B* gene has a 1-bp mismatch (*). PCR product was directly sequenced, and the peaks were analysed to detect mutated sequences. The deleted sequence was underlined and shaded in gray. sgRNA and PAM sequences are denoted in red and blue, respectively.

For Peer Review Only

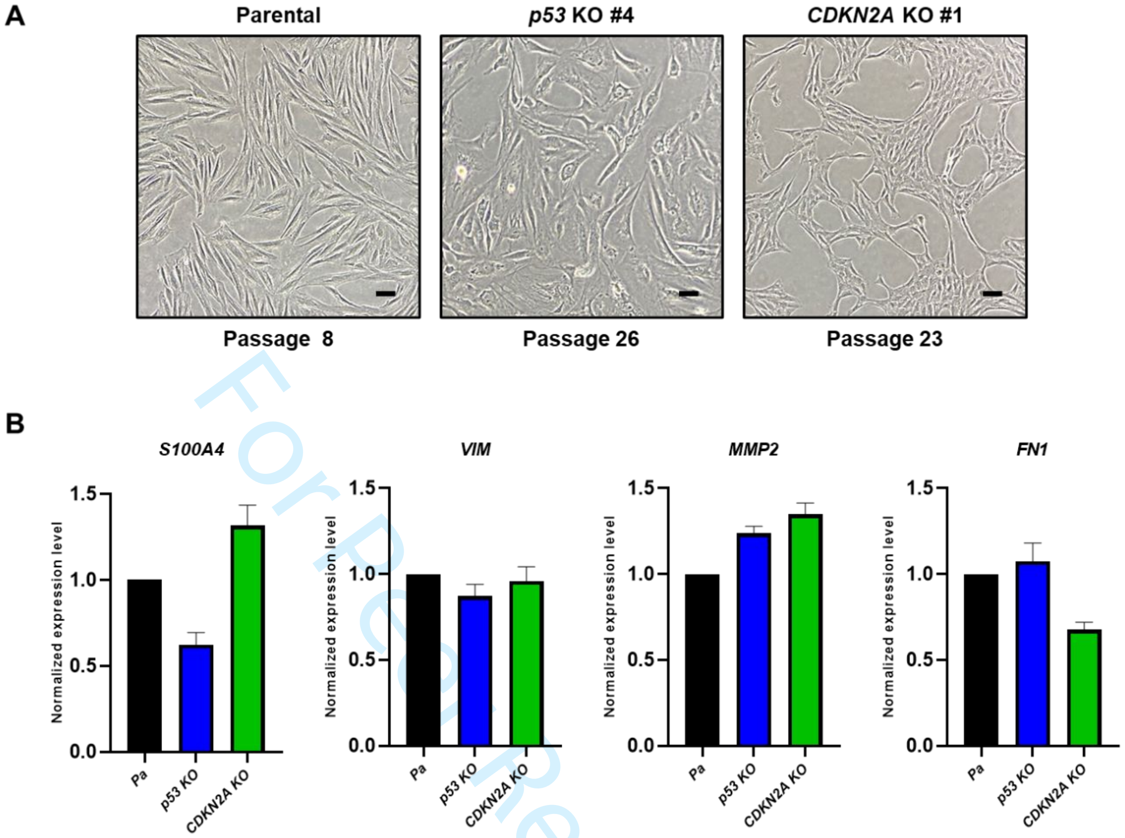

Figure S6 Morphologies and expression of fibroblast markers in primary and immortalized marmoset cells. (A) Cellular morphology. Each cell line was seeded at 5 × 10^5 cells/60 mm dish. Parental and *CDKN2A*$^{-/-}$ clone #1 were photographed at day 3. Fresh medica was added to *p53*$^{-/-}$ cells (clone #4) on day 3, and cells were photographed on day 4. Scale bars, 50 μm. (B) Expression levels of fibroblast marker genes including *S100 calcium binding protein A4 (S100A4), vimentin (VIM), matrix metallopeptidase 2 (MMP2)* and *fibronectin 1 (FN1)*. Expression levels were measured by the quantitative real-time RT-PCR. Pa, parental.

For Peer Review Only

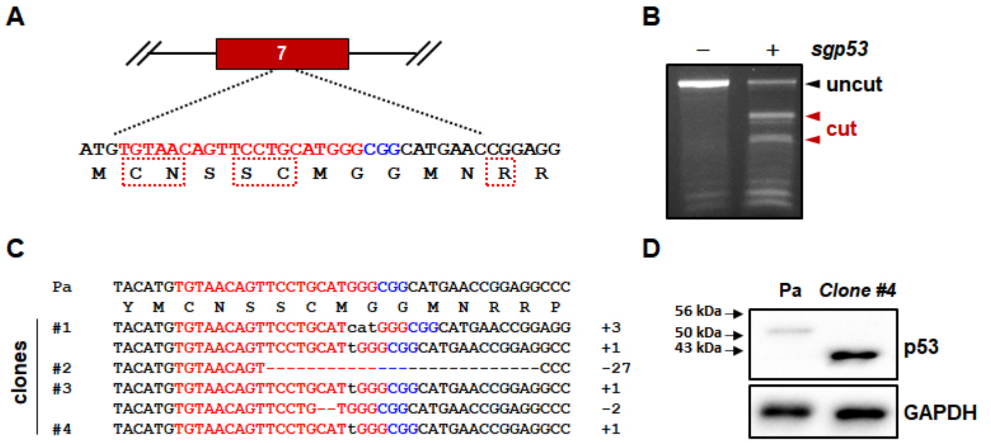

Figure 1. Cas9-mediated p53 gene targeting in primary skin fibroblasts isolated from a common marmoset monkey. (A) Schematic representation of the *p53*-specific sgRNA on exon 7. Target and PAM sequences are denoted by red and blue colors, respectively. Dotted boxes indicate the amino acids critical for the DNA-binding function of p53 protein as a transcription factor. (B) Endonuclease activity induced by *p53*-specific sgRNA (sgp53). After infection with lentivirus expressing both Cas9 and *p53*-specific sgRNA, Cas9-induced indel mutations was examined using genomic DNA samples from parental primary skin fibroblasts (−) and puromycin-selected cells (+) by T7E1 assay. (C) Mutated p53 sequences observed in the mutant cell clones. − denotes deleted nucleotides; sequences in lower case denote nucleotide insertions. (D) Western blot analysis of p53 proteins in primary skin fibroblasts (parental; Pa) and p53$^{-/-}$ skin fibroblasts (clone #4 in c).

208x95mm (96 x 96 DPI)

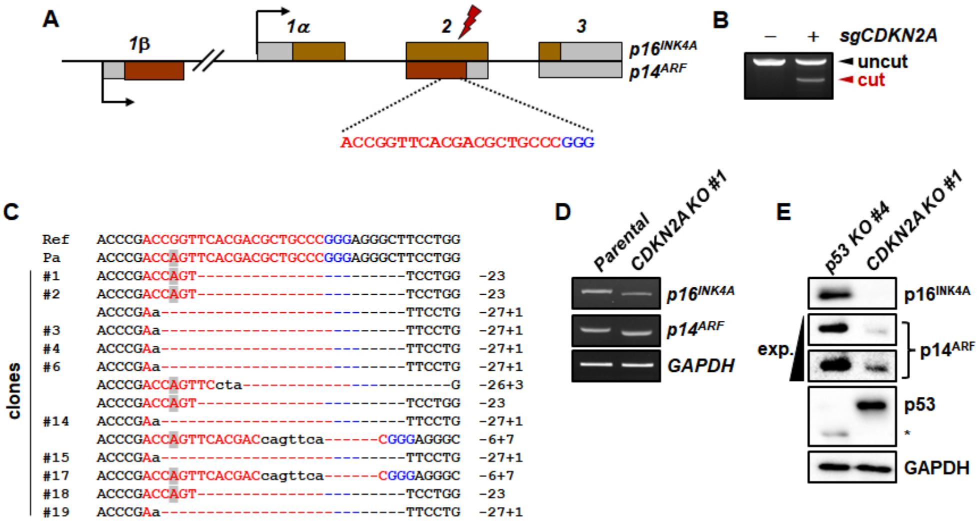

Figure 2. Generation of mutant marmoset skin fibroblasts deficient in both $p16^{INK4A}$ and $p14^{ARF}$ genes using CRISPR-Cas9. (A) Schematic representation of CDKN2A-specific sgRNA on exon 2, which is common to both $p16^{INK4A}$ and $p14^{ARF}$. Target and PAM sequences are denoted by red and blue colors, respectively. (B) Endonuclease activity of Cas9 induced by common exon 2-specific sgRNA (sgCDKN2A). T7E1 assays were conducted using genomic DNA samples from parental primary skin fibroblasts (−) and puromycin-selected cells (+) after infection with lentivirus expressing both Cas9 and sgCDKN2A. (C) Mutated CDKN2A sequences observed in the clones. A polymorphic nucleotide is shaded in gray. Ref, reference genomic DNA sequence of the CDKN2A locus; Pa, parental marmoset skin fibroblasts; − denotes deleted nucleotides; sequences in lower case denote nucleotide insertions. (D) Western blot analysis of $p16^{INK4A}$ and $p14^{ARF}$ proteins in $p53^{-/-}$ cells (Fig. 1 clone #4) and $CDKN2A^{-/-}$ cells (clone #1 in c). Short exp., short exposure time; long exp., long exposure time.

233x125mm (96 x 96 DPI)

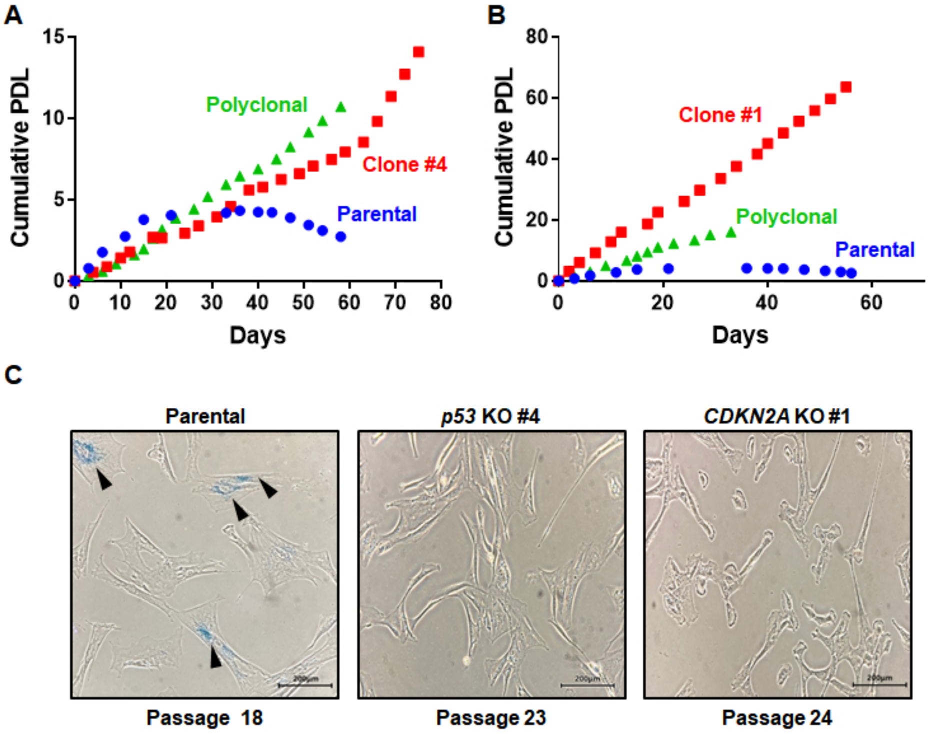

Figure 3. Immortalized phenotypes observed in both p53- and CDKN2A-deficient marmoset skin fibroblasts. (A, B) Cumulative population doublings (PDs) of *p53*-deficient (A) and *CDKN2A*-deficient (B) marmoset skin fibroblasts. Growth of polyclonal cells (purple triangles) and monoclonal mutant cells (red rectangles; $p53^{-/-}$ clone #4 and $CDKN2A^{-/-}$ clone #1) were compared with that of parental primary skin fibroblasts (blue circle). (C) Senescence-associated β-galactosidase (SAβ-gal) assays conducted with marmoset skin fibroblasts and immortalized *p53*- and *CDKN2A*-deficient clones at the denoted passages. Scale bar, 200 μm.

208x156mm (96 x 96 DPI)

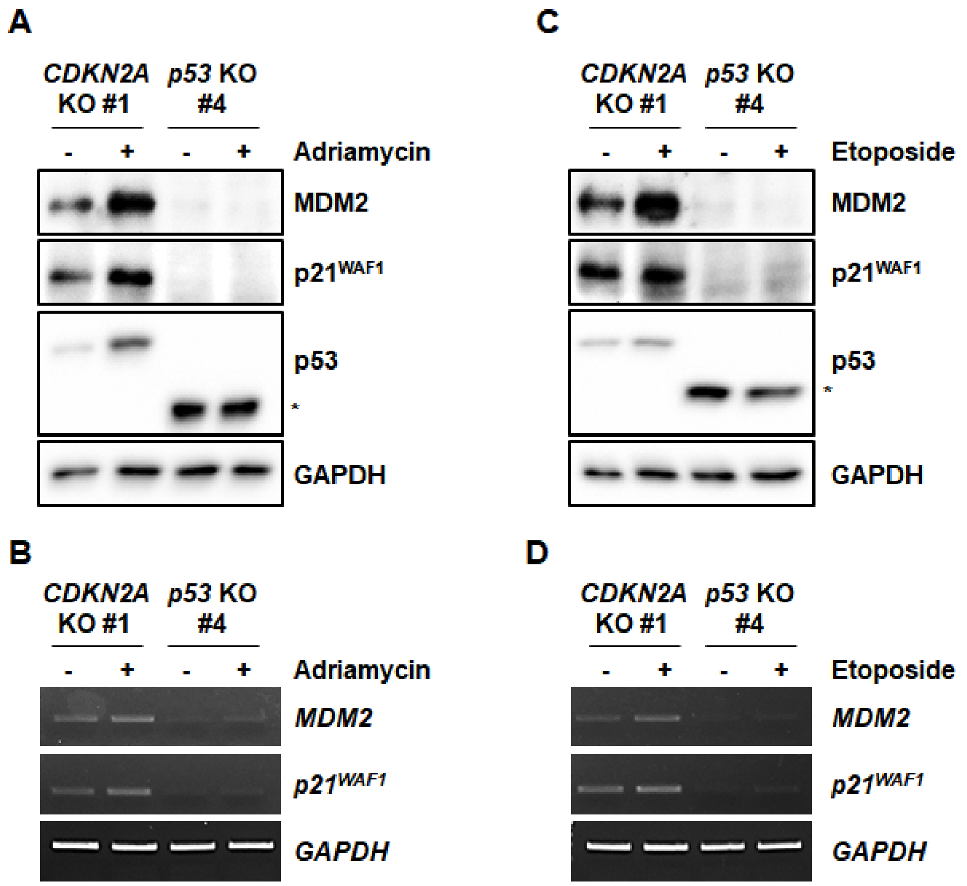

Figure 4. Induction of p53 target genes by genotoxic stresses in immortalized *CDKN2A*-deficient marmoset skin fibroblasts. (A) Western blot analysis of p53 and its target gene products, MDM2 and p21^{WAF1}, in immortalized *p53*- and *CDKN2A*-deficient marmoset skin fibroblasts treated with 200 ng/ml adriamycin for 4 hours. (B) Assessment of adriamycin-induced p53 target gene (*MDM2* and *p21^{WAF1}*) transcription by semi-quantitative RT-PCR. Immortalized marmoset cells were treated as in a. The asterisk indicates truncated marmoset p53 proteins. (C) Western blot analysis of p53 and its target gene products, MDM2 and p21^{WAF1}, in immortalized *p53*- and *CDKN2A*-deficient marmoset skin fibroblasts treated with 0.5 μM etoposide for 4 hours. The asterisk indicates truncated marmoset p53 proteins. (D) Etoposide induction of p53 target gene (*MDM2* and *p21^{WAF1}*) transcription in immortalized marmoset cells. Semi-quantitative RT-PCR reactions were conducted with cells treated as in c.

165x148mm (96 x 96 DPI)
